# Clinical utilisation of adenosine stress CMR and its influence on patient management in a tertiary cardiac centre

**DOI:** 10.1186/1532-429X-15-S1-P270

**Published:** 2013-01-30

**Authors:** Neha Sekhri, Adam Timmis, Anjum Mahmood, Eva C Sammut, Mark A Westwood, Ceri Davies, Saidi A Mohiddin, Roshan Weerackody, Andrew Wragg, Dan Jones, Redha Boubertakh, Steffen E Petersen, Anthony Mathur

**Affiliations:** 1 London Chest Hospital, London, UK

## Background

Recent landmark studies like the MR-IMPACT and CE-MARC have demonstrated the advantage of stress CMR over other imaging modalities in ischaemia detection of coronary artery disease. However to date there are no studies demonstrating how stress CMR influences patient management in clinical practice.

## Methods

A retrospective cohort of 2139 patients who had undergone both adenosine stress CMR (2008-2011) and a coronary angiographic procedure (2006 to 2011) was identified. 142 patients with a diagnosis of cardiomyopathy and 98 patients with previous CABG were excluded. 1899 patients comprised the study group with the latest date of followup being 31.8.2012. Data was extracted from each patient's hospital health record and analysed using STATA version 8.0. This study was undertaken as part of clinical service evaluation.

## Results

Indications for stress CMR were: symptomatic chest pain (74%), assessment of functional significance of residual coronary disease (12%), assessment of LV function (12%) and nonspecified reasons (2%). Inducible perfusion defects were identified in 1546 (81%) patients among whom 534 (35%) had had prior angiographic assessment. 224 patients underwent percutaneous intervention (PCI) post CMR with the majority performed in those with perfusion defects (92%, n=206). In these 206 patients, the perfusion defect matched the territory of the stented coronary vessel in 89% (187) patients. The median time to PCI was 77 days (IQR 37-134) in the matched defect group and 157 days (IQR 79-488) in the unmatched defect group, p<0.003. On follow up (1.6±0.85years), 72% (n=149) denied any symptoms, 18% (n=38) had ongoing symptoms and 9% (n=19) had had a repeat revascularisation procedure. 2% (n=5) patients died during follow up.

## Conclusions

We have demonstrated that stress CMR can both guide percutaneous intervention and influence patient management. To our knowledge, this is the first study demonstrating not only the diagnostic utility of stress CMR but also its integration into contemporary clinical practice. Further research is needed to determine how it can further improve patient outcomes.

## Funding

None

